# Which Neural Network to Choose for Post-Fault Localization, Dynamic State Estimation, and Optimal Measurement Placement in Power Systems?

**DOI:** 10.3389/fdata.2021.692493

**Published:** 2021-08-31

**Authors:** Andrei Afonin, Michael Chertkov

**Affiliations:** ^1^ Department of Intelligent Information Systems and Technologies, Moscow Institute of Physics and Technologies, Moscow, Russia; ^2^ Program in Applied Mathematics, University of Arizona, Tucson, AZ, United States

**Keywords:** neural networks, physics-informed machine learning, power system, fault localization, state estimation

## Abstract

We consider a power transmission system monitored using phasor measurement units (PMUs) placed at significant, but not all, nodes of the system. Assuming that a sufficient number of distinct single-line faults, specifically the pre-fault state and the (not cleared) post-fault state, are recorded by the PMUs and are available for training, we first design a comprehensive sequence of neural networks (NNs) locating the faulty line. Performance of different NNs in the sequence, including linear regression, feed-forward NNs, AlexNet, graph convolutional NNs, neural linear ordinary differential equations (ODEs) and neural graph-based ODEs, ordered according to the type and amount of the power flow physics involved, are compared for different levels of observability. Second, we build a sequence of advanced power system dynamics–informed and neural ODE–based machine learning schemes that are trained, given the pre-fault state, to predict the post-fault state and also, in parallel, to estimate system parameters. Finally, third and continuing to work with the first (fault localization) setting, we design an (NN-based) algorithm which discovers optimal PMU placement.

## 1 Introduction

The essence of this manuscript is in addressing classic problems in power systems (PSs)—state estimation (Schweppe and Wildes, 1970; Monticelli, 1999; Baalbergen et al., 2009; Zhao et al., 2019), fault detection and localization (Jiang et al., 2014; Xie et al., 2014), and optimal phasor measurement unit (PMU) placement (Yuill et al., 2011; Yue Zhao et al., 2012; Li et al., 2019)—using the new machine learning tools. Specifically, we consider the following two settings which are relevant for the transmission level PS monitoring of faults which are not cleared but which are also not system critical, that is, which result in the post-fault transient, typically occurring in the course of 5–20 s and leading to a post-fault steady state which is distinct from the pre-fault steady state.^1^

• I) Given a set of samples, each consistent with 1) a pre-fault state and 2) a post-fault state, both recorded at the nodes of the system equipped with PMUs, and 3) a faulty line that is identified/localized^2^, we aim to find a function which predicts the post-fault state (at the PMU locations), that is, maps a mismatch between 1) and 2), considered as an input, to 3), considered as an output.
• II) The same as the above in (I) but not utilizing 3) and aiming at finding a universal dynamical model which maps 1) to 2)^3^.
• III) Given a limited budget on the number of active PMUs available for the system monitoring (which is normally a fraction of all of the system’s nodes), and given an algorithm available for (I) above which can be applied to any PMU placement, we aim to find the optimal placement of PMUs.

While addressing the outlined problems, we will not only present a modern algorithmic machine learning solution but also suggest, for each of the problems, a number of solutions/algorithms organized in a sequence. The sequence(s) will be ordered, according to the amount and type of the power flow information used in this particular solution. Therefore, the first algorithm(s) in the sequence will be PS-physics-agnostic, that is, dealing with the PMU data as it would with any other data stream. We will see that these algorithms may be quite successful in the regime(s) of the routine fault which is not stressing the entire PS too much and/or in the regime of a very detailed PMU coverage, when all or almost all nodes of the system are monitored. On the other extreme of the spectrum, we will be discussing very demanding regime(s) when either the fault is severe or observability is very limited, or possibly both. In this high-stress regime, we expect the PS-agnostic schemes to perform very poorly and will thus be focusing on injecting some (not all) PS-guidance into the algorithm. In general, we are interested in building a road map toward fault detection, localization, and interpretation, which would help the system operator to have a choice of a wide variety of tools, to select one from depending on the current operational needs.

Application of ML to the problems related to localization of the faulty line (I) in our list of the tasks above and, also, the challenge of the PMU placement for better detection were already discussed in the study by (Li et al., 2019), which is thus a starting point for our analysis. Specifically, (Li et al., 2019) proposed a method to identify the faulted line based on a convolutional neural network (CNN) classifier using bus voltages and suggested a placement strategy for PMUs under varying uncertain conditions, system observability, and measurement quality. This manuscript is linked to the study by (Li et al., 2019) in a number of ways, some already mentioned above, but also we continue to work in here with the same data source and the same model.

We generate data using the power system toolbox (Chow and Cheung, 1992) and work throughout the manuscript with the same exemplary model—the IEEE 68-bus electrical network (see Section 3.1 for details). We apply similar measures of performance, for example, the cross-entropy (Wikipedia, 2021a) loss function to solve the classification problem of fault localization in Section 2.1 and the mean squared error (MSE) (Wikipedia, 2021b) loss function as we solve the regression problem of the dynamic state estimation in Section 2.2 and the classification problem of the optimal PMU placement in Section 2.3.

As mentioned above, in this manuscript, we describe machine learning (ML) models juxtaposed against each other and experimented with in the following sections to establish their regime of optimal use. Our aim is four-fold. First, we want to make the description of the models simple and transparent. Second, we attempt to clarify the logic behind the models’ design/architecture, focusing, in particular, on explaining why particular models are chosen to answer the power system learning problems (failure localization and/or state estimation/prediction). Third, we build the hierarchy of models, in the sense that models introduced earlier are used as building blocks to construct more advanced models introduced later in the section. Finally, fourth, the hierarchy of models will also be gauged and commented on in terms of the level of physics of the underlying power system processes involved in their construction.

## 2 Materials and Methods

### 2.1 Detection of Failure in the Static Regime

This section is split into subsections as follows. We remind the reader of the basic elements of the machine learning (ML) architecture and training in Section 2.1.1. We also use it to set the stage for other learning problems considered in the following sections. The experimental setup of the manuscript is detailed in Section 3.1. Linear regression (LR), the feed-forward neural network (FF-NN), AlexNet, and graph convolution neural networks (GC-NNs) are introduced in Sections 2.1.2, 2.1.3, 2.1.4, 2.1.5. In Section 4.1, we present and discuss the results of our failure detection experiments with the NNs (and also other NNs related to neural ODEs, as described in Section 2.2).

#### 2.1.1 Parameterization and Training

A supervised ML model is introduced as a map of the input, normally denoted as *x*, to the output, normally denoted as *y*, which is parameterized by the vector of parameters, *ϕ*. We use the notation ML_*ϕ*_ : *x* → *y* and emphasize that the ML model is of a general position. In the supervised learning setting, which we are mainly focusing on in this manuscript, we are given *I* samples of the input/output data, *i* = 1, … , *I*:*x* ^(*i*)^, *y* ^(*i*)^, which we also call incidental *I* samples.

In the fault localization classification problem, aiming to detect a failed line, we follow the scheme of (Li et al., 2019). We input a sample vector, $x_{V_{o}}=\left(x_{a}|a\in V_{o}\right)$, with as many components as the number of observed nodes, where $V_{o}$ is the set of observed nodes of the power system network. Here, $V_{0}$ is a subset of the set of all nodes of the network, $V_{0}\subset V$. The output, $y=\left(y_{ab}|\left\{a,b\right\}\in E\right)$, is the vector of the dimensionality equal to the number of power lines in the system (number of edges, E, in the power network, where each line is connecting two neighboring nodes of the network). Each output vector is sparse, with only one nonzero (unity) element corresponding to the location of the fault.

A popular choice (see, e.g., (Li et al., 2019)) of the loss function for the case of a classification output, for example, of the fault localization of interest here, is the so-called cross-entropy (CE) loss function (Wikipedia, 2021a), which is as follows:

$$\begin{array}{l} L_{\text{ CE}}\left(\phi ;V_{o}\right)=-\frac{1}{I}\overset{I}{\underset{i=1}{\sum }}\underset{\left\{a,b\right\}\in E}{\sum }y_{ab}^{\left(i\right)}\log\left({\text{ML}}_{\phi ;ab}\left(x_{V_{o}}^{\left(i\right)}\right)\right), \end{array}$$ (1)

where ${\text{ML}}_{\phi ;ab}\left(x_{V_{o}}^{\left(i\right)}\right)=y_{\phi ;ab}^{\left(i\right)}$ shows the {a,b}∈E component of the output vector for the *i*th sample generated by the NN function with the fixed vector of the parameters, *ϕ*; the sums in Eq. 1 correspond to averaging over the empirical probability associated with *I* actual (true) observations of the faults at specific locations within the grid.

The process of training the ML model becomes to solve the following optimization problem:

$$\begin{array}{l} \phi_{\text{trained}}\left(V_{o}\right)≐\text{arg}\underset{\phi }{\min}L_{\text{ CE}}\left(\phi ;V_{o}\right), \end{array}$$ (2)

where arg min means finding the argument of the minimum with respect to the vector of parameters, *ϕ*, and $L_{\text{ CE}}\left(\phi ;V_{o}\right)$ is defined in Eq. 1. It should be noticed that the result (2) depends on the set of the observed nodes, $V_{o}$.

#### 2.1.2 Linear Regression

Linear regression (LR) is the simplest ML model, which is the benchmark for comparison in all of our experiments. If it performs well in a regime, other models will not be needed. It is also appropriate to mention that in the case of a small-to-mild perturbation, power systems are well explained by linear equations (static or dynamic), therefore providing additional (even though imprecise) legitimacy to the LR.

Formally, the LR model maps the input vector, $x\in R^{n}$, to the output vector, $y\in R^{s}$, according to *y* = *Wx* + *b*, where $W\in R^{s\times n}$ and $b\in R^{s}$ are, respectively, the multiplicative matrix and the additive vector to be learned. *ϕ≐*(*W*, *b*) append *W* and *b* in one vector of parameters. We will also use the following (standard in the ML literature) notation for the linear map:

$$\begin{array}{l} {\text{LR}}_{\phi }:\;x\to y=Wx+b. \end{array}$$ (3)

The fault localization version of the LR learning consists in solving Eqs 1, 2 with the generic function ML_*ϕ*_ substituted by LR_*ϕ*_.

#### 2.1.3 Feed-Forward Neural Network With Two Layers

The feed-forward neural network (FFNN) with two layers is one of the simplest architectures of nonlinear NNs. We use it in the regime of limited observability when we expect that due to the severity of the perturbation, the LR reconstruction may not be sufficient. The FFNN is implemented with the rectified linear unit (ReLU) sandwiched by two LR layers as follows:

$$\begin{array}{l} {\text{FFNN}}_{\phi }:\;x\to {\text{LR}}_{\phi }\to \text{ReLU}\to {\text{LR}}_{\phi }\to y, \end{array}$$ (4)

where *ϕ* is the vector of parameters on the left side that is built by appending *W* and *b* parameters of the two *LR* layers on the right (parameters associated with the two layers are independent), and therefore, if $x\in R^{n}$ is the input vector and $y\in R^{s}$ is the output vector (as in the LR case), then *p* is the dimension of the hidden ReLU layer (notice that the ReLU layer is fixed, that is, there are no parameters associated with the layer). Training of the FFNN_*ϕ*_ is, like before in the case of the LR_*ϕ*_, reduced to solving Eqs 1, 2 with the generic function ML_*ϕ*_ substituted by FFNN_*ϕ*_.

#### 2.1.4 AlexNet Convolutional Neural Network

AlexNet (Krizhevsky et al., 2012) is a convolutional neural network (CNN) which was cited the most in the ML literature. It was used in many other applications as a starting CNN option, in particular for the real-time faulted line detection reported in the study by (Li et al., 2019). Following (Li et al., 2019), we adapt here the classic AlexNet layout. We use the 13-layer AlexNet CNN to reconstruct line failures. The CNN takes input at the observed nodes and output status of lines (in the form of the sparse vector with unity at the position of the failure). The CNN has four convolutional layers and one fully connected layer. Every convolutional layer consists of the convolution sublayer and the max-pooling sublayer. Training of the network to localize the fault requires solving Eqs 1, 2 with the generic function ML_*ϕ*_ substituted by the AlexNet_*ϕ*_.

#### 2.1.5 Graph Convolutional Neural Network

The graph convolutional neural network (GCNN) is an NN which we build by making relations between variables in the (hidden) layers based on the known graph of the power system. In this regard, the GCNN is informed, at least in part, about the physical laws and controls associated with the power system operations. Specifically, we utilize a sparse *n* × *n* matrix, |*Y*|, built from the absolute values of the impedances associated with power lines connecting *n* nodes of the system to each other, in constructing the GCNN (the matrix is sparse because the degree of a typical node in a transmission-level power system is somewhere in the 1–4 range). We follow the construction of (Kipf and Welling, 2016) and use *Y* to build the convolutional layer of the GCNN. Let *H* be the input vector to the graph convolutional layer, then the output *f* (*H*, *A*) of such a layer is $f\left(H,A\right)=\sigma \left(D^{-\frac{1}{2}}AD^{-\frac{1}{2}}HW\right)$, where *W* is a matrix of parameters; *A* = |*Y*| + *I*, where *I* is the unit matrix and *σ*() is a nonlinear activation function. We normally use ReLU() for *σ*(). *D* is the diagonal matrix built from the vector of the node degrees within the power system graph. $D^{-\frac{1}{2}}$ stands for the matrix derived from *D* by taking the component-wise inverse square root. We use GC_*ϕ*_ for the GC operation where *ϕ* denotes all the parameters needed to describe the graph convolution map from the *n*-dimensional input to the *p*-dimensional vector representing the hidden layer. With a minor abuse of notations, the resulting map becomes as follows:

$$\begin{array}{l} {\text{GCNN}}_{\phi }:\;x\to {\text{GC}}_{\phi }\to \text{ReLU}\to {\text{LR}}_{\phi }\to y, \end{array}$$ (5)

where $x\in R^{n}$ is the input vector to our model, $y\in R^{s}$ is its output, and GC_*ϕ*_(*x*) is the *p*-dimensional vector of the intermediate layer *p*. As always, two independent vectors of parameters on the right-hand side of Eq. 5 are appended into the resulting vector of the parameters on the right-hand side of Eq. 5. Training of the GCNN_*ϕ*_ to localize the fault is reduced to solving Eqs 1, 2 with the generic function ML_*ϕ*_ substituted by the GCNN_*ϕ*_.

### 2.2 Dynamic Models

This section is devoted to the introduction and discussion of the dynamic models, transitioning to the models gracefully from the topic (of fault localization) discussed in the preceding section. We show that the dynamic models can be used both in the context of static and dynamic state estimation, where in the former case, the dynamic component of the model helps to provide input about (otherwise hidden) dynamic aspects of the underlying phenomena. We start with the discussion of a generic, and thus power system (PS)-physics-agnostic, neural ODE model in Section 2.2.1 and then start to add the PS-physics in steps progressing to the physics-informed neural networks (PINNs) in Section 2.2.2, to the Hamiltonian neural networks (HNNs) in Section 2.2.3, and, finally, to the direct ODE NN based on the swing equations in Section 2.2.4.

#### 2.2.1 Neural Ordinary Differential Equations

The neural ODE is a modern NN method suggested in the study by (Chen et al., 2018). It builds an input-to-output map as if it would come from the temporal dynamics governed by the parameterized ODE as follows:

$$\begin{array}{l} t\in \left[0,T\right]:\;\frac{dx\left(t\right)}{dt}=f_{\phi }\left(x\left(t\right)\right), \end{array}$$ (6)

where *ϕ* is a (possibly time-dependent) vector parameterizing the “rhs” of the ODE, that is, *f*_*ϕ*(*t*)_ [*x*(*t*)], using a NN. It is assumed that an ODE solver, taking *f* as an input, can be used in a black-box fashion to train the NN. When considered in discrete time, Eq. 6 becomes *k* = 1, … , *K*, *t*_*k*_ = Δ*k*, Δ = *T*/*K* as follows:

$$\begin{array}{l} x\left(t_{k+1}\right)=x\left(t_{k}\right)+\Delta f_{\phi }\left(x\left(t_{k}\right)\right), \end{array}$$ (7)

where Δ is the time step. Neural ODEs are also naturally linked to the so-called ResNet (residual network) architecture discussed in the study by (He et al., 2015). Consistent with notations used for other models, we have the following:

$$\begin{array}{l} {\text{NeuralODE}}_{\phi }:\;x\left(0\right)\to x\left(T\right), \end{array}$$ (8)

where $x\left(0\right)\in R^{n}$ is the input vector to our model, and $x\left(T\right)\in R^{n}$ is the output which is of the same dimensionality, *n*, as the input. We will work in the following with an LR version of *f*_*ϕ*_ and with a graph CNN version of *f*_*ϕ*_ in Eq. 6, and then replace NeuralODE in Eq. 8 by LinODE and GraphODE, respectively, where LinODE and GraphODE mean that *f*_*ϕ*(*t*)_ [*x*(*t*)] is parameterized by the linear layer and the graph convolutional layer, correspondingly. To make the output of the LinODE and GraphODE versions of Eq. 8 consistent with the output of other (static) models discussed so far, we will additionally map *x*(*T*) to *y*, as discussed above, inserting the additional ReLU function (we remind the reader that *y* is the output vector which, in the training stage, has only one nonzero component correspondent to the faulty line). We therefore add, as already discussed in Section 2.1, the LinODE and GraphODE augmented with the ReLU function to the list of other (static) schemes resolving the task of the failed line localization.

However, we may also consider NeuralODE (8) as a part of the dynamic state estimation (DSE) scheme. In this case, we assume that *x*(*T*) is the observed output and then we may train the NeuralODE by minimizing the following:

$$\begin{array}{ll} & \text{arg}\underset{\phi }{\min}L_{2\text{;NeuralODE}}\left(\phi \right),\;L_{2\text{;NeuralODE}}\left(\phi \right)= \end{array}$$ (9)

$$\begin{array}{ll} & \overset{I}{\underset{i=1}{\sum }}{\left\|x^{\left(i\right)}\left(T\right)-{\text{NeuralODE}}_{\phi }\left(x^{\left(i\right)}\left(0\right)\right)\right\|}^{2}. \end{array}$$ (10)

Moreover, we may generalize Eq. 8 and consider the entire trajectory, which we will also call “the path,” $\left\{x\left(t\right)|t\in \left.\right]0,T\left.\right]\right\}$, or (more realistically) its discretized version, {*x* (*t*_*k*_)|*k* = 1, … , *K*}, as the output of the NeuralODE_*ϕ*_, which is as follows:

$$\begin{array}{l} {\text{Path}-\text{NeuralODE}}_{\phi }:\;x\left(0\right)\to \left\{x\left(t_{k}\right)|k=1,\cdots \;,K\right\}. \end{array}$$ (11)

Then the exemplary training problem—finding the best (functional) map in the path version of the NeuralODE—becomes the following:

$$\begin{array}{ll} & \text{arg}\;\underset{\phi }{\min}L_{2\text{;Path}-\text{NeuralODE}}\left(\phi \right),\;L_{2\text{;Path}-\text{NeuralODE}}\left(\phi \right)=\\ & \text{arg}\underset{\phi }{\min}\;\;\overset{I}{\underset{i=1}{\sum }}\;\;\frac{1}{K}\;\;\overset{K}{\underset{k=1}{\sum }}\|x^{\left(i\right)}\left(t_{k}\right)\;-\;{\text{Path}-\text{NeuralODE}}_{\phi }\left(x^{\left(i\right)}\left(0\right);t_{k}\right){\|}^{2}. \end{array}$$ (12)

As will be argued in the remaining subsections of this section, we may project the formulation of Eqs 11, 12 to the problems of interest to the power system dynamics. Specifically, we may consider *x*(*t*) corresponding to dynamics of the state of the power system measured as a function of time at the observed mode (e.g., *S*(*t*) and/or *V*(*t*)) in the transient regime. In this case, the training data, that is, {*x* ^(*i*)^ (*t*)|*i* = 1, … , *I*, *t* ∈ [0, *T*]}, can be generated by a dynamic power flow solver, resolving many more degrees of freedom (at many more nodes) and, therefore, producing results much slower than the trained Path-NeuralODE reduced model.

#### 2.2.2 Physics-Informed Neural Net

The structure of the so-called physics-informed NN (PINN) is described in the study by (Raissi, 2018). It is based on some early ideas on tuning an NN to satisfy the output of a differential equation (Lagaris et al., 1998). We are seeking to use it for data fitting of a concrete version of the ODE model (6), with *f*_*ϕ*_[*x*(*t*)] replaced by *f*_*ψ*_[*x*(*t*)], which is specified by “physics,” where *ψ* thus stands for the vector of physics-meaningful (explainable or interpretable) parameters, as follows:

$$\begin{array}{l} \frac{dx\left(t\right)}{dt}=f_{\psi }\left(x\left(t\right)\right), \end{array}$$ (13)

where *x*(*t*) stands for measurements changing in time *t*. We built a neural network, mapping *t* to ${\hat{x}}_{\phi }\left(t\right)$. We aim to search through the space of *ϕ* to minimize the difference between ${\hat{x}}_{\phi }\left(t\right)$ and the actual measurements, *x* at the time *t*. In the PINN of the study by (Raissi, 2018), the goal is achieved by minimizing the following loss function:

$$\begin{array}{ll} & \underset{\phi ,\psi }{\mathrm{arg min}}L_{PINN},\;L_{PINN}\left(\phi ,\psi \right)=\lambda \overset{K}{\underset{k=1}{\sum }}{\left({\hat{x}}_{\phi }\left(t_{k}\right)-x\left(t_{k}\right)\right)}^{2}\\ & +\overset{K}{\underset{k=1}{\sum }}{\left({\hat{x}}_{\phi }\left(t_{k+1}\right)-{\hat{x}}_{\phi }\left(t_{k}\right)-\Delta f_{\psi }\left(t_{k},{\hat{x}}_{\phi }\left(t_{k}\right)\right)\right)}^{2}, \end{array}$$ (14)

where over *ϕ* represents the aforementioned NN, and also over *ψ*, which may be represented by a NN, or can also include some “physical” parameters, that is, parameters which allow physical (the power system in our case) interpretation ^4^; *λ* is a pre-set hyper-parameter; the entire data path, {*x*(*t*)}_*K*_ = {*t*_*k*_, *x*_*k*_|*k* = 1, … , *K*}, is assumed to be known.

A number of technical and terminology remarks are in order. First, the vector of physical parameters, which may describe *ψ* or its part, should be tuned to the specifics of the power system, and this is what will be done below in Sections 2.2.3, 2.2.4. Second, generalization of the scheme from the ODE to the PDE is straightforward. In fact, the Burgers PDE was the enabling example in the study by (Raissi, 2018).

Finally, third, let us also mention that the PINN ideas (Raissi, 2018) are similar to the approach known under the name of the learning differential equation (LDE) (see, e.g., (Bento et al., 2010) and references therein) and are also discussed in the context of learning power system dynamics in the study by (Lokhov et al., 2017). The similarity between the two approaches is in the form of the loss function, including differential equations *via* the *l*_2_-term and the also kind of similar, but not identical, *l*_1_ regularization term. The difference between the PINN approach of (Raissi, 2018) and the LDE approach of (Bento et al., 2010) is two-fold. On one hand, no NNs were used in the study by (Bento et al., 2010) to represent unknown functions, while embedding NNs into the scheme is the main novelty of the study by (Raissi, 2018). On the other hand, the LDE approach of the study by (Bento et al., 2010) consisted in learning the stochastic differential equations and, specifically, the unknown physical parameters, *ψ*, in *f*_*ψ*_(*t*, *u*) (if we use the extension of the PINN) just introduced above in the first remark. The stochastic component revealed itself in the study by (Bento et al., 2010) *via* the appearance of the inverse covariance matrix (also called the precision or concentration matrix), which may also be considered as contributing, in full or partially, the vector of the physics-meaningful training parameters, *ψ*. Finally, fourth, let us also mention that the PINN scheme of the study by (Raissi, 2018) was adapted to dynamic parameter learning in the power system setting in the study by (Misyris et al., 2019). See also the related discussion below in Section 2.2.4.

#### 2.2.3 Hamiltonian Neural Net

As already mentioned above, more structures related to our understanding (or expectation) about the physics of the problem can be embedded into the NeuralODE and PINN. Specifically, if the underlying ODE is of a conservative (Hamiltonian) type, we can construct what is coined in the studies by (Zhong et al., 2020a; Zhong et al., 2020b) as the Hamiltonian NN. However, the system of equations describing the power system dynamics (which are yet to be introduced) is not conservative, therefore suggesting that a more general model than the bare Hamiltonian one can be appropriate here. It seems reasonable to consider the dynamical system described by the so-called port-Hamiltonian system of equations (van der Schaft et al., 2006) as follows:

$$(\begin{array}{c} \dot{\text{q}}\\ \dot{\text{p}} \end{array})=\left(\left(\begin{array}{cc} 0 & \text{I}\\ -\text{I} & 0 \end{array}\right)-{\text{D}}_{\phi }\left(p,q\right)\right)\left(\begin{array}{c} \frac{\partial H_{\phi }\left(p,q\right)}{\partial \text{q}}\\ \frac{\partial H_{\phi }\left(p,q\right)}{\partial \text{p}} \end{array}\right)+\left(\begin{array}{c} 0\\ {\text{F}}_{\phi }\left(p,q\right) \end{array}\right),$$ (15)

where the coordinate vector, *p*, and the momentum vector, *q*, are of the same dimensionality, *m*, I is the *m* × *m*-dimensional identity matrix, *H*_*ϕ*_ (*p*, *q*) is the Hamiltonian function, D_*ϕ*_ (*q*) is the symmetric positive-definite *m* × *m* dissipation matrix (function), and *F*_*ϕ*_ (*p*, *q*) is the source function.

Obviously, one may consider Eq. 15 as a particular case of the general ODE Eq. 6 where *x* = (*p*, *q*). Then one can naturally introduce the (port-) Hamiltonian version of the Path-Neural ODE, substituting Path-NeuralODE_*ϕ*_ in Eq. 11 by path-HNN_*ϕ*_, and then train it by minimizing Eq. 12 where the respective substitution is also made.

#### 2.2.4 Direct Ordinary Differential Equation Neural Network Based on the Swing Equations

A popular model of the power system, extending the static power flow equations to the dynamical case, is the so-called nonlinear and dissipative swing equations governing dynamics of the phase of the voltage potential, *θ*_*a*_(*t*), as follows:

$$\begin{array}{ll} & \forall a\in V:\;m_{a}{\ddot{\theta }}_{a}+d_{a}{\dot{\theta }}_{a}=P_{a}-\;\;\;\;\;\;\underset{b\in V;\left\{a,b\right\}\in E}{\sum }\;\;\;\;\;\beta_{ab}v_{a}v_{b}\sin\left(\theta_{a}\;-\;\theta_{b}\right)\\ & -\underset{b\in V;\left\{a,b\right\}\in E}{\sum }g_{ab}v_{a}\left(v_{a}-v_{b}\cos\left(\theta_{a}-\theta_{b}\right)\right), \end{array}$$ (16)

where *v*_*a*_ is the absolute value of the voltage potential at a node, *a*, *β*_*ab*_ and *g*_*ab*_ are the susceptance and conductance of the line {*a*, *b*}, defined as imaginary and real parts of the line admittance, *Y*_*ab*_ = *g*_*ab*_ + *iβ*_*ab*_, and *m*_*a*_ and *d*_*a*_ are inertia and the so-called droop coefficients at the node *a* ^5^.

Normally (in the power system literature), the power network and line characteristics in Eq. 16 correspond to the actual physical lines; then line parameters, *m*, *d*, are dynamic physical parameters associated with devices’ (generators and loads) inertia and damping (also frequency control), respectively, while *β*, *g* are static physical parameters of the respective lines and devices. Here, we adapt this physical picture to a reduced model of power systems. This adaptation is obviously blurred by limited observability. We assume, extending the static consideration of the study by (Pagnier and Chertkov, 2021), that Eq. 16 also applies to a reduced set of nodes where the PMU devices are located and measurements are available. In this setting, we consider a complete graph that connected the observed nodes and do not assume that the (effective) nodal and line parameters are known—instead we aim to learn the effective parameters. It should also be noticed that Eq. 16 can be viewed as a particular, that is, more structured, version of the port-Hamiltonian system of Eq. 15. Here, like in the case of Path-HNN, we introduce the direct ODE NN (DIRODENN) version of the Path-Neural ODE, substituting Path-NeuralODE_*ϕ*_ in Eq. 11 by Path-DIRODENN_*ϕ*_, and then train it by minimizing Eq. 12 where the respective substitution is also made.

### 2.3 Machine Learning Algorithms for Optimal Placement of Phasor Measurement Units

As the first set of experiments (detection of failure in the static regime, reported and discussed in Section 2.1) show, accuracy of the ML model varies very significantly not only on the percentage of nodes where observations are available but also on where exactly within the system the observations are made. This dependency motives the third set of experiments discussed below. Specifically, we focus in this section on building ML schemes which are capable of discovering locations for close to optimal placement of the phasor measurement units (PMUs) for the given level of observability efficiently, that is, fast.

It should be noticed that this problem of searching for the optimal PMU placement was already addressed in the study by (Li et al., 2019). However, the algorithm suggested that there was “passive,” which means that the algorithm worked in parallel with the training of the main model (in the setting of our first experiment). Stating it differently, in the passive search, the placement configurations do not utilize the information received so far. In theory, this passive sampling conducted without a feedback loop should eventually find the optimal PMU placement; however, the passive search normally takes a long time.

In the following part, we develop an active strategy which reinforces the search by taking advantage of the measurement made so far, thus allowing a much faster discovery of the optimal PMU placement than in the passive case considered so far.

**FIGURE 1:**
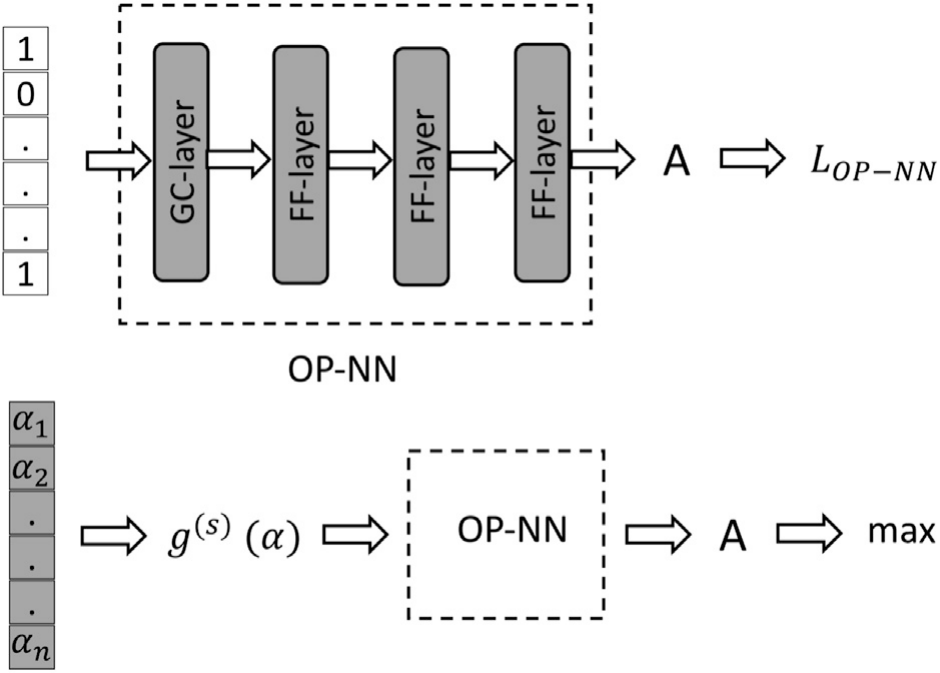
Architecture of the optimal placement NN. Top and bottom sub-figures illustrate the two-step training of the OP-NN. Gray shading highlights components of the architecture trained during the respective stage (parameters are tuned). See text for details.

**FIGURE 2:**
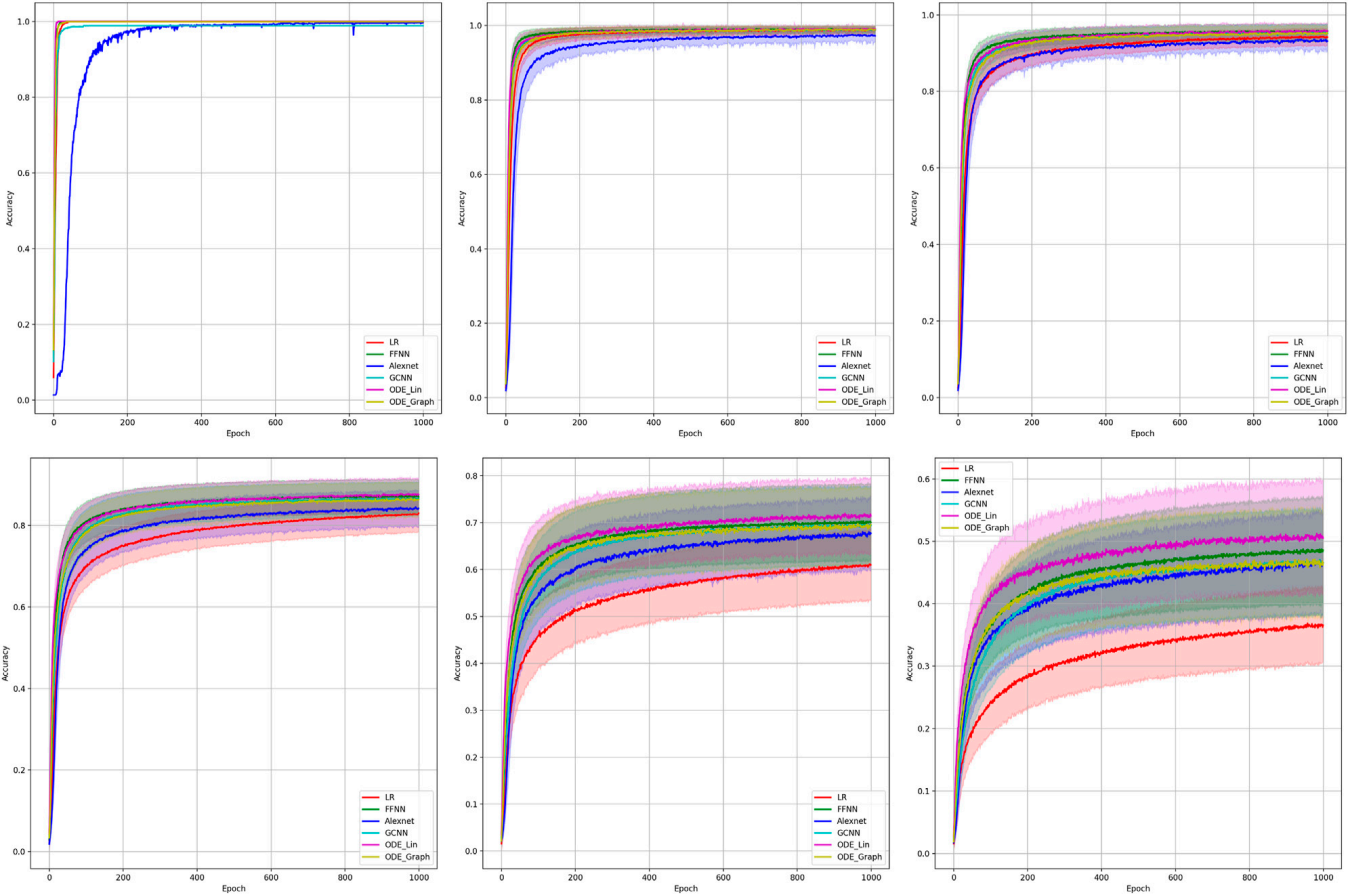
Comparison of the learning model performance for detection of line failure in the static regime. Sub-figures correspond to **(left-to-right and top-to-bottom)** 100, 70, 40, 20, 10, and 5% of observability.

The main idea of the approach, illustrated in Figure 1, is in solving the OP problem in two steps: first, find a function which maps each set of observed nodes to a score expressing the accuracy, *A*, of the reconstruction, $f:V_{o}\to A$, where *A* ∈ (0, 1), and 0 and 1 correspond to the complete failure and success of the reconstruction, respectively. Second, find the argument of the minimum of the function, suggesting the desired optimal placement (OP). We construct the function, *f*, by means of learning from multiple input–output–placement (IO-P) samples, where each IO-P sample aggregates multiple samples correspondent to experiments discussed in Section 2.1 that are conducted for the same placeemnt (i.e., the same set of observed nodes, $V_{o}$) and for the same basic NN model, for example, the LR model. Accuracy, *A*, of a particular OP-IO sample, corresponding to the asymptotic y-axis value of a curve in Figure 2 achieved at the end of the training run, becomes the output of the OP-NN, as shown in the left sub-figure of Figure 1. Additional details on the structure of the OP-NN are given below. Parameters of the OP-NN, built from four layers (a graph convolutional layer, followed by three feed-forward layers), are trained during the first stage by minimizing *L*_OP-NN_, chosen to be the *l*_2_ norm between *A*-predictions and *A*-observations. The second stage consists in fixing parameters of the OP-NN and then finding the arg-maximum of the resulting optimal function, *f*. It is achieved by finding the optimal vector $\alpha =\left(\alpha_{a}\in R|a\in V\right)$, built from n=|V| real-valued components, mapped *via* *g* ^(*s*)^ (*α*) ∗ OP-NN to the accuracy, *A*. Here, the *g* ^(*s*)^ (*α*) is the function mapping a real-valued *α* associated to a vector of the same length *n* having nonzero components at the nodes of the suggested PMU placement; formally, this is as follows:

$$\begin{array}{l} g_{a}^{\left(s\right)}\left(\alpha \right)\;=\;\frac{\exp\left(\alpha_{a}\right)}{\underset{b\in V}{\sum }\exp\left(\alpha_{b}\right)}\times \left\{\;\;\begin{array}{cc} 1, & \alpha_{a}\in \text{ top}-s\text{ comp. of }\alpha ;\\ 0, & \text{otherwise.} \end{array}\right. \end{array}$$

This additional “softening” function allows us to take advantage of the automatic differentiation for finding the minimum of *f* efficiently.

We also use the transfer learning technique (Zhuang et al., 2019) to speed up and improve the quality of the OP scheme. Specifically, we first implement the scheme on (by far) the fastest, but also the least accurate, linear regression (LR) method and then use the pre-trained LR-OP-NN as a warm-start for training other (more accurate, but slower) methods of the OP reconstruction.

## 3 Results

### 3.1 Detection of Failure: Experiments

We are conducting our experiments on the ground truth data, (*x*, *y*), generated using the power system toolbox (Chow and Cheung, 1992) on the exemplary IEEE 68-bus electrical network, consisting of *n* = 68 nodes and *m* = 87 lines.

We follow the supervised learning setup of the study by (Li et al., 2019), which is as follows:

• The power network is described in terms of the known, symmetric (*n* × *n*) admittance matrix with 2*m* off-diagonal nonzero complex elements.
• We limit our analysis to single-line failures. To generate the ground truth data, we pick the failed line i. i. d. at random from the m=|E| options. The fault is permanent (not cleared); however, we assume that it is operating in the so-called *N*−1 safe regime, with the system stabilized after any of the single-line faults to a new steady state (thus achieved in the regime of the corrected admittance matrix derived from the initial admittance matrix by removing a line, that is, forcing the admittance of the corrected line to zero).
• Observations, before and after the fault, are available at $\left|V_{o}\right|$ nodes assumed to be equipped with phasor measurement units, or alternative measurement equipment. We consider the cases with 5%, 10%, 20%, 40%, 70%, and 100% of observed nodes. Creating the initial training dataset, we pick the observed nodes at random. For each setting of the observed nodes, we train each of the ML models (yet to be described). We repeat the training protocol 50 times for each ML model in each case of partial observability and then present the averaged results.
• Input (sample): *x* is generated using the power system toolbox (PST) (Chow and Cheung, 1992) according to *x* = *Y*Δ*U*, where *Y* ∈ *C* ^*n* × *s*^ is a *n* × *s*, where n=|V| and $s=|V_{o}|$, the sub-matrix of the full (*n* × *n*) admittance matrix, and Δ*U* ∈ *C* ^*s*^ is the complex valued vector of changes, that is, difference in readings before and after the incident, in the voltage potentials at the observed nodes. Here, we assume that *Y* is known. It should be noticed that each component of the *x*-vector is complex, and therefore represented in the NN modeling *via* two real channels.
• Output (sample): m=|E| is the binary vector of the empirical line failure probability, $\forall \left\{a,b\right\}\in E:\;y_{ab}\in \left\{0,1\right\},\sum_{\left\{a,b\right\}\in E}y_{ab}=1$. In our experiments, each of the 50 samples corresponds to a new randomly removed line of the system.

The AlexNet model is presented in Table 1 and details on the architectures of other models are as follows:

$$\begin{array}{ll} & \text{LR}:\;x\to \text{Lin}\left(68,87\right)\to y\\ & \text{FFNN}:\;x\to \text{Lin}\left(68,32\right)\to ReLU\to \text{Lin}\left(32,87\right)\to y\\ & \text{GCNN}:x\;\to \;\text{GraphConv}\left(68,32\right)\;\to \;\text{ReLU}\;\to \;\text{Lin}\left(32,87\right)\;\to \;y\\ & \text{ODENN}:\text{ODEBlock}\left(68,68\right)\to \text{Lin}\left(68,87\right)\to y \end{array}$$

where ODEBlock = [Linear (input size, output size)], followed by the ReLU in the case of the LinODE and ODEBlock = [GraphConv (input size, output size), followed by the ReLU in the case of the GraphODE.

**TABLE 1:** AlexNet (Krizhevsky et al., 2012) architecture. Abbreviations are inp.,input; out.,output; ker.,kernel; chan.,channels.

Conv (inp. Size = 68, inp. chan. = 1, out. chan. = 4, ker. size = 5, stride = 1)
ReLU
MaxPool (ker. size = 2, stride = 2)
Conv (inp. chan. = 4, out. chan. = 8, ker. size = 5, stride = 1)
ReLU
MaxPool (ker. size = 2, stride = 2)
Conv (inp. chan. = 8, out. chan. = 8, ker. size = 3, stride = 1)
ReLU
MaxPool (ker. size = 2, stride = 2)
Conv (inp. chan. = 8, out. chan. = 8, ker. size = 3, stride = 1)
ReLU
MaxPool (ker. size = 2, stride = 2)
Linear (inp. size = 16, out. size = 87)

Training consists in minimizing the cross-entropy loss function (Wikipedia (2021a). Cross, 2021a) with respect to the vector of parameters, *ϕ*, over 1,000 epochs. We use the Adam (Kingma and Ba, 2014) gradient optimization method with the learning rate 1*e*−3 and the value of the *l*_2_ regularization 5*e*−6.

Results of our experiments are shown in Figure 2 and Table 2. Color-shaded regions show the distribution of the learning curves in terms of their dependence on the random choice of the observable nodes. These regions are obtained by sampling at random, given the percentage of nodes and learning on the data these nodes provide. For each of the models, a bold curve represents the mean over the sampling. The mean curve is seen to be located within the color-shaded (sampled) region. Discussion of the results is presented in Section 4.1.

**TABLE 2:** Summary of the detection failure experiments. The columns show the following: models; quality of performance under 5% observability and 0% SNR; number of parameters; time per epoch (in sec), averaged over 1,000 epochs for the CPU and GPU, respectively.

Model	Quality	# Param	*t*_CPU_	*t*_GPU_
LR	0.4993	6,003	0.016	0.023
FFNN	0.6490	5,079	0.021	0.018
AlexNet	0.6229	2,071	0.100	0.048
GCNN	0.6342	5,079	0.029	0.019
ODE Lin	**0.6737**	10,695	1.238	1.847
ODE Graph	0.6398	10,695	1.284	1.791

### 3.2 Dynamic State Estimation: Experiments

It seems appropriate to start the discussion of our dynamic state estimation (DSE) experiments reported in this section from a clarification on the use of terms. The subject of the DSE has a long, distinguished, and continuing history in power systems (see, e.g., the most recent report of the IEEE Task Force on Power System Dynamic State and Parameter Estimation (Zhao et al., 2019) and references therein). The essence of DSE is in introducing a dynamical equation, for example, corresponding to one of the dynamic models described in the preceding Section 2.2, and then reconstructing/learning coefficients in the equation from the data.

**FIGURE 3:**
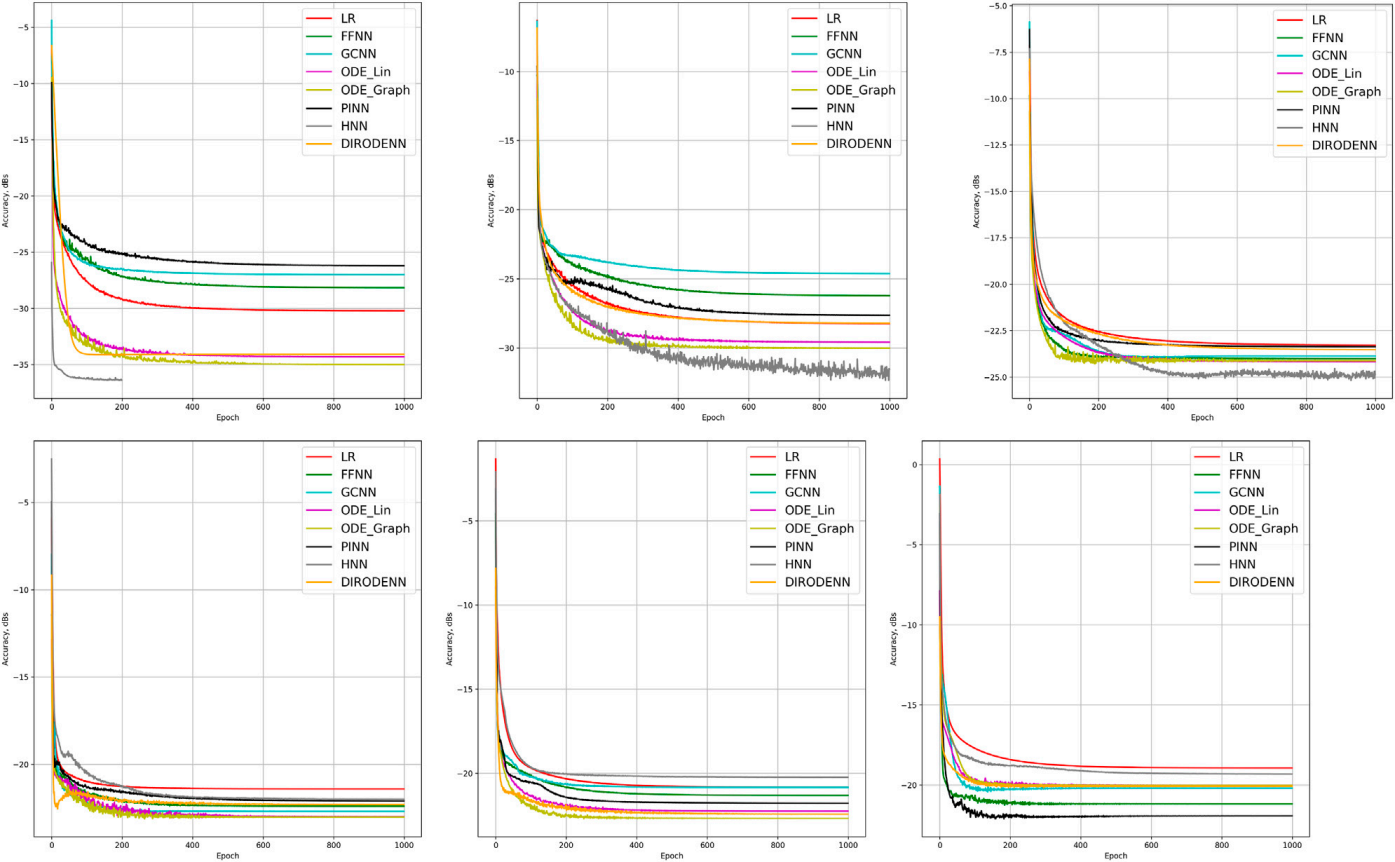
Comparison of the learning model performance for detection of line failure in the dynamic regime. Sub-figures correspond to (left-to-right and top-to-bottom) 100, 70, 40, 20, 10, and 5% of observability.

As in the (static) fault detection setting, described in Section 3.1, we are conducting our dynamic experiments with the data generated in the power system toolbox (Chow and Cheung, 1992) on the IEEE 68-bus electrical network under sufficiently small dynamic perturbations^6^. Changes in the dynamic setting (when compared with the static one of Section 3.1) are as follows. Input/output is the dynamic path, {*x*(*t*)}_*K*_, where at each *t*_*k*_, *x* (*t*_*k*_) represents the voltage potential (the absolute value and phase) measured at the observed nodes of the system. That is, ${\left\{x\left(t\right)\right\}}_{K}\in R^{2\times 68\times K}$, in the case of the full observability and, ${\left\{x\left(t\right)\right\}}_{K}\in R^{2\times |V_{o}|\times K}$, in the case of the partial observability. We are experimenting with (5%, 10%, 20%, 40%, 70%, and 100%) node observation levels. We experiment with the dynamic models expressing different degrees of physics, discussed in Section 2.2, but also test static models adapted to the (time-incremental) map^7^. In this case of the dynamic state estimation, we select observation nodes at random and then repeat multiple experiments (collect statistics) for this particular set of the observed nodes. Our training consists in minimizing the *l*_2_ norm given by Eq. 12, adapted, respectively, to different dynamical models considered ^8^. Actual optimization is implemented using the Adam (Kingma and Ba, 2014) gradient method over 1,000 epochs (under exception of the case of the HNN model under 100% observability, where the training is over 200 epochs) with the optimal learning rate and the value of the *l*_2_ regularization presented in Table 3 for each model. Tables 4, 5, 6, 7, 8, 9 show details on the results on the comparison of the dynamic state estimation (CDSE) experiments under 100, 70, 40, 20, and 10% of observability. These tables show the comparison of the best loss (in decibels), number of model parameters, and required CPU time. Also, one can see the results in Figure 3. The performance of prediction (in dB) is accessed according to the log of the ration of the mismatch between the predicted and the observed, normalized to the observed: $\text{Accuracy}=10\mathrm{lg}\left(\frac{P_{\text{error}}}{P_{\text{output}}}\right)$, where $P_{\text{error}}=\sum_{t}\|x_{t}^{\left(\text{pred}\right)}-x_{t}^{\left(\text{pred}\right)}{\|}_{2}^{2}$, $P_{\text{output}}=\|x_{t}^{\left(\text{pred}\right)}{\|}_{2}^{2}$.

**TABLE 3:** Summary of the optimal hyper-parameters (the optimal rate | value of the *l*_2_ regularization) in the dynamic state estimation experiments.

Model	100%	70%	40%
LR	1e-3 | 3e-7	1e-3 | 3e-7	1e-3 | 3e-7
FFNN	1e-2 | 1e-6	1e-2 | 1e-6	1e-2 | 1e-7
GCNN	1e-3 | 5e-8	5e-3 | 5e-8	5e-3 | 5e-9
AlexNet	1e-3 | 3e-7	- | -	- | -
ODE Lin	1e-2 | 1e-8	1e-2 | 1e-8	1e-2 | 1e-8
ODE Graph	2e-2 | 3e-9	2e-2 | 3e-9	3e-2 | 3e-9
PINN	1e-2 | 3e-9	1e-2 | 3e-8	5e-3 | 3e-8
HNN	1e-2 | 0	3e-3 | 0	3e-3 | 0
DIRODENN	5e-3 | 1e-8	5e-3 | 1e-8	1e-2 | 1e-8
**Model**	**20%**	**10%**	**5%**
LR	1e-3 | 3e-7	1e-3 | 3e-7	1e-3 | 3e-7
FFNN	2e-2 | 5e-7	1e-2 | 5e-8	1e-2 | 5e-8
GCNN	1e-2 | 5e-6	1e-2 | 3e-6	5e-2 | 5e-8
AlexNet	- | -	- | -	- | -
ODE Lin	5e-2 | 5e-8	5e-2 | 5e-8	5e-2 | 5e-8
ODE Graph	5e-2 | 5e-9	5e-2 | 5e-9	2e-2 | 0
PINN	5e-3 | 8e-5	5e-3 | 8e-5	5e-3 | 8e-5
HNN	3e-3 | 0	5e-3 | 0	1e-2 | 0
DIRODENN	5e-2 | 1e-8	5e-2 | 1e-8	5e-2 | 1e-8

**TABLE 4:** CDSE models under 100% observability.

Model	Loss	# Param	*t*_CPU_
LR	−30.21	4,692	0.010
FFNN	−28.16	4,452	0.012
GCNN	−26.98	4,452	0.016
AlexNet	−19.10	6,800	0.058
ODE Lin	−34.30	4,692	0.301
ODE Graph	−**34.98**	4,692	0.378
PINN	−26.19	4,452	0.349
HNN	−**36.39**	37,469	1.860
DIRODENN	−34.10	204	0.457

**TABLE 5:** CDSE models under 70% observability.

Model	Loss	# Param	*t*_CPU_
LR	−28.24	3,196	0.026
FFNN	−26.20	3,748	0.029
GCNN	−24.61	4,452	0.032
ODE Lin	−29.58	5,358	0.362
ODE Graph	−**30.02**	6,370	0.564
PINN	−27.63	3,748	0.376
HNN	−**32.34**	29,899	2.140
DIRODENN	−28.21	12,786	0.513

**TABLE 6:** CDSE models under 40% observability.

Model	Loss	# Param	*t*_CPU_
LR	−23.28	1,836	0.025
FFNN	−24.01	3,108	0.029
GCNN	−23.93	4,452	0.031
ODE Lin	−24.18	2,538	0.305
ODE Graph	−**24.29**	3,630	0.456
PINN	−23.52	3,108	0.381
HNN	−**25.18**	12,799	1.626
DIRODENN	−23.52	7,286	0.412

**TABLE 7:** CDSE models under 20% observability.

Model	Loss	# Param	*t*_CPU_
LR	−21.41	952	0.024
FFNN	−22.39	2,692	0.035
GCNN	−22.75	4,452	0.032
ODE Lin	−**23.05**	1,134	0.268
ODE Graph	−**23.11**	1,849	0.378
PINN	−22.10	2,692	0.381
HNN	−21.99	5,116	1.427
DIRODENN	−22.54	3,711	0.356

**TABLE 8:** CDSE models under 10% observability.

Model	Loss	# Param	*t*_CPU_
LR	−20.82	476	0.023
FFNN	−21.30	2,468	0.027
GCNN	−20.82	4,452	0.031
ODE Lin	−22.24	518	0.202
ODE Graph	−**22.69**	890	0.242
PINN	−21.77	2,468	0.398
HNN	−20.23	2,099	1.369
DIRODENN	−**22.42**	1,786	0.330

**TABLE 9:** CDSE models under 5% observability.

Model	Loss	# Param	*t*_CPU_
LR	−18.92	272	0.023
FFNN	−**21.24**	2,372	0.027
GCNN	−20.40	2,372	0.029
ODE Lin	−20.06	479	0.181
ODE Graph	−20.03	479	0.228
PINN	−**22.09**	2,372	0.404
HNN	−19.30	1,046	1.268
DIRODENN	−20.14	961	0.314

### 3.3 Optimal Placement of Phasor Measurement Units: Experiments

We pre-train the OP function, *f*, illustrated in Figure 1 on the LR training data in the experimental setting of Section 2.1, on 1,600 samples, each characterized by the (placement nodes and LR accuracy) pair. Architecture of the OP function, *f*, is shown in Table 10. The results are used as a warm-start for training all other schemes (the FFNN, GCNN, AlexNet, ODE Lin, and ODE Graph) independently and each on 350 samples (placement nodes and method accuracy). Specifically, in training the advanced methods, we fix parameters of the first three layers according to the pre-trained LR-OP-NN and retrain the last layer. We use the Adam (Kingma and Ba, 2014) gradient method for 1,200 epochs with an initial step of 0.08 and decrease it by a factor of 10 every 300 epochs at the pre-training (LR) stage. We use the same method for 300 epochs, with an initial step of 0.01 and decrease it by a factor of 10 times every 100 epochs at the post-training (advanced methods) stage.

**TABLE 10:** Additional details on the architecture of the optimal placement neural network (OP-NN) from Figure 1.

Optimal placement neural network:
GraphConv layer (68, 16)
ReLU
FF layer (16, 16)
ReLU
FF layer (16, 16)
ReLU
FF layer (16, 6)
Sigmoid

**FIGURE 4:**
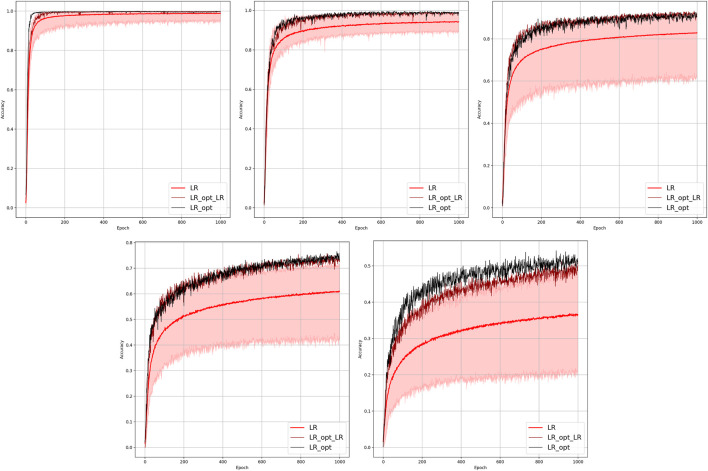
Comparison of the placement learning with the LR model for different observabilities. Sub-figures correspond to **(left-to-right and top-to-bottom)** 70, 40, 20, 10, and 5% of observability.

**FIGURE 5:**
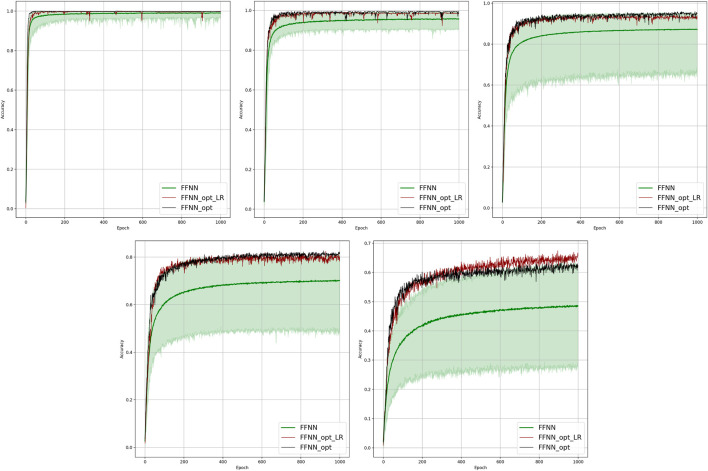
Comparison of the placement learning with the FFNN model for different observabilities. Sub-figures correspond to **(left-to-right and top-to-bottom)** 70, 40, 20, 10, and 5% of observability.

**FIGURE 6:**
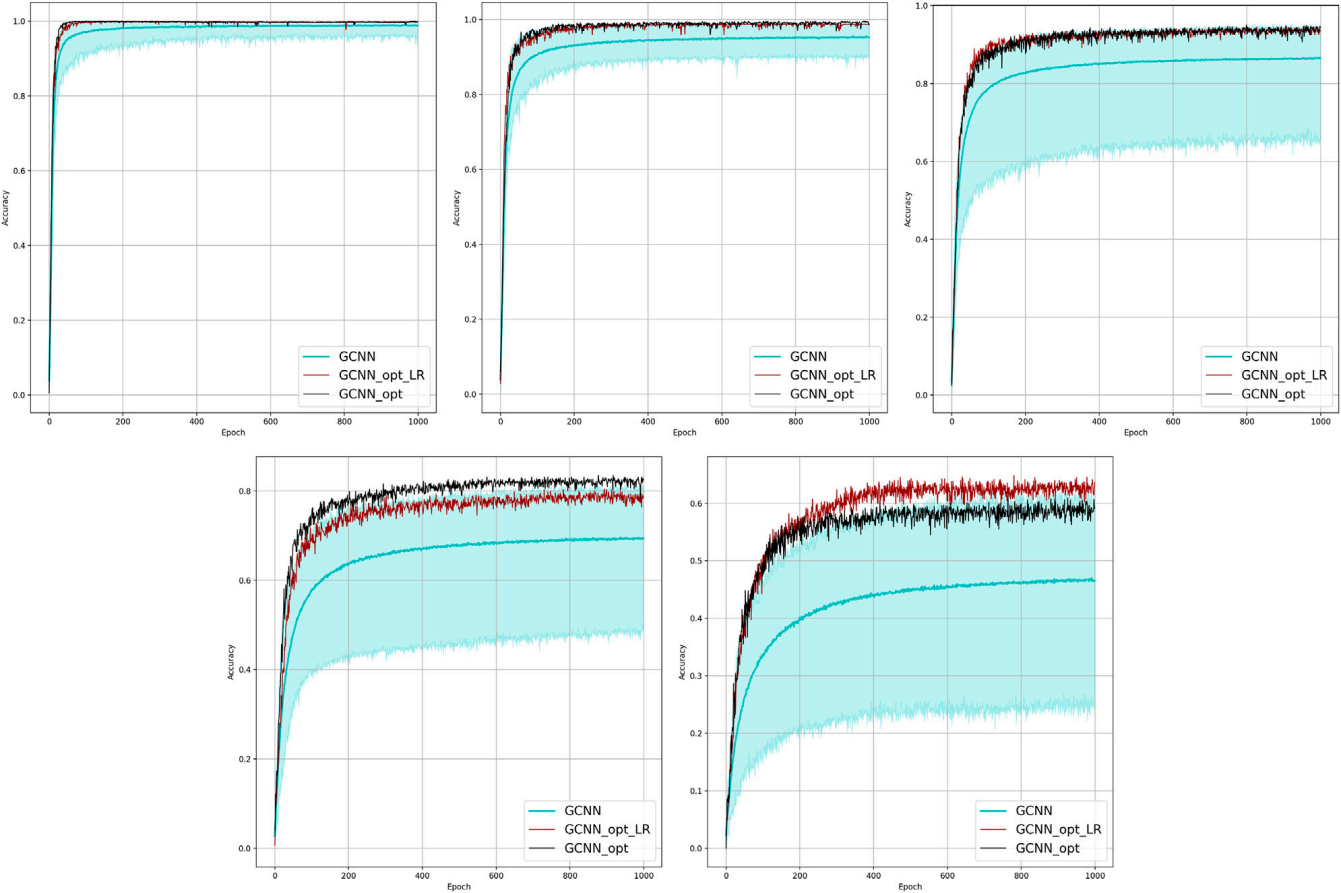
Comparison of the placement learning with the GCNN model for different observabilities. Sub-figures correspond to **(left-to-right and top-to-bottom)** 70, 40, 20, 10, and 5% of observability.

**FIGURE 7:**
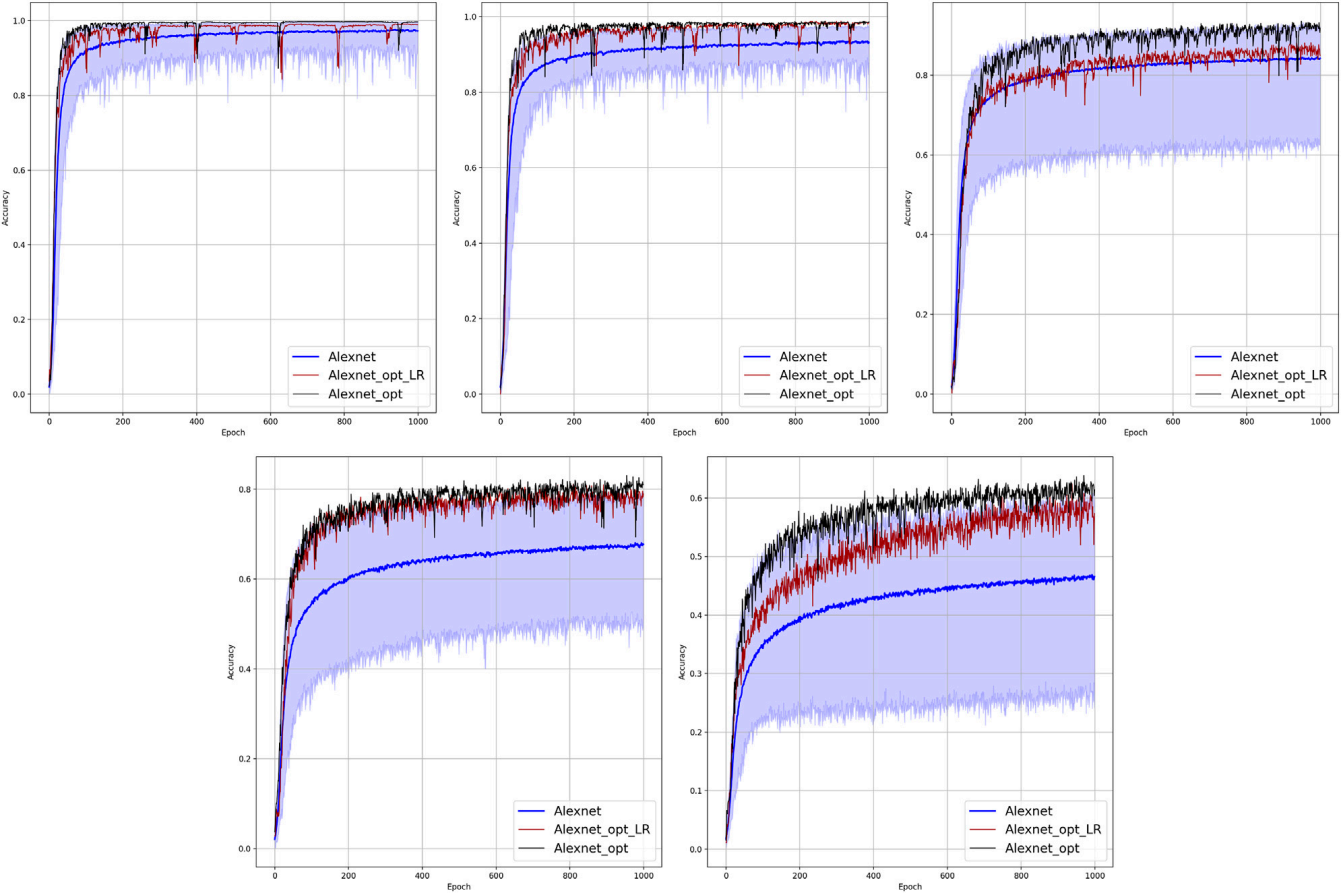
Comparison of the placement learning with the AlexNet model for different observabilities. Sub-figures correspond to **(left-to-right and top-to-bottom)** 70, 40, 20, 10, and 5% of observability.

**FIGURE 8:**
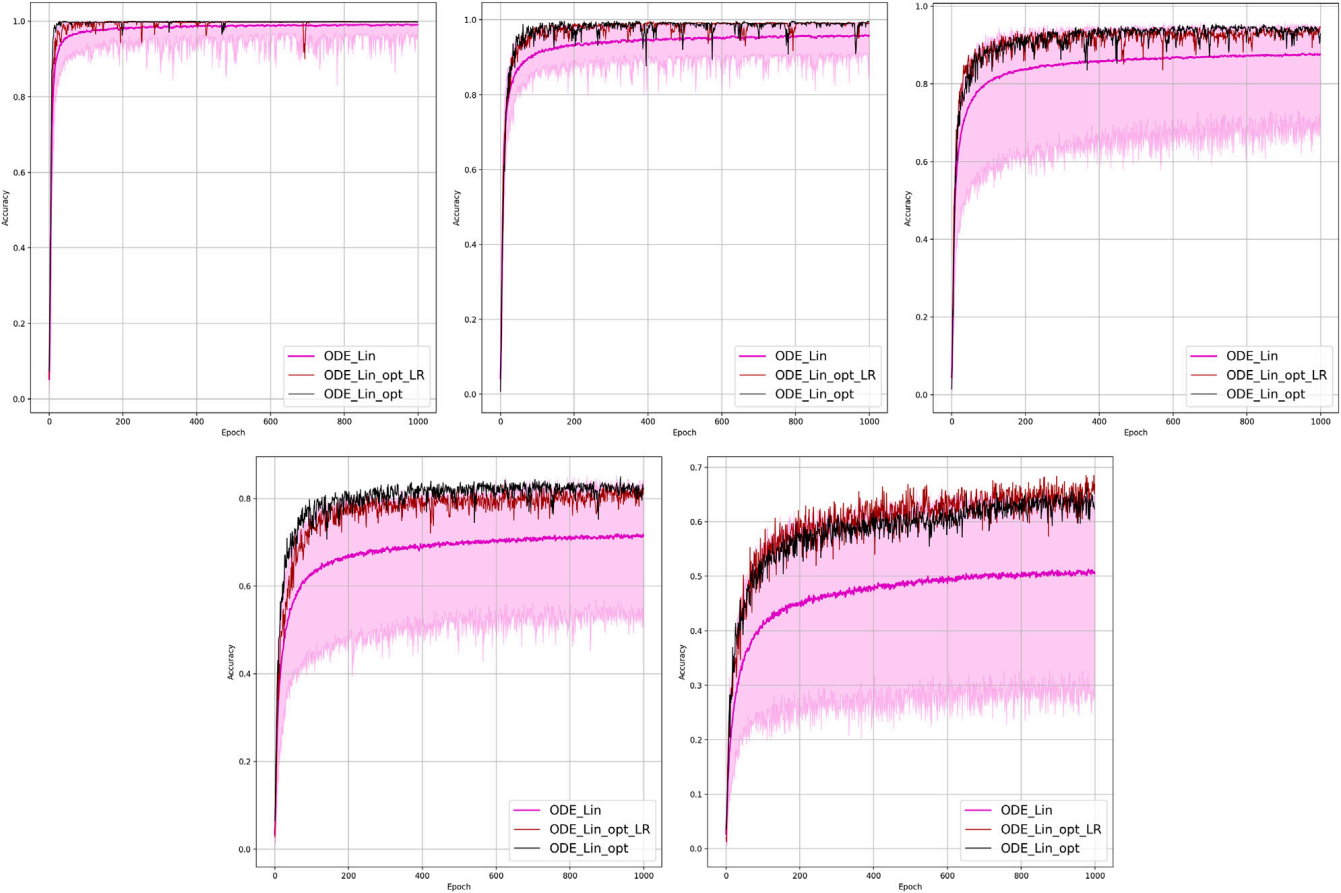
Comparison of the placement learning with the Linear ODE model for different observabilities. Sub-figures correspond to **(left-to-right and top-to-bottom)** 70, 40, 20, 10, and 5% of observability.

**FIGURE 9:**
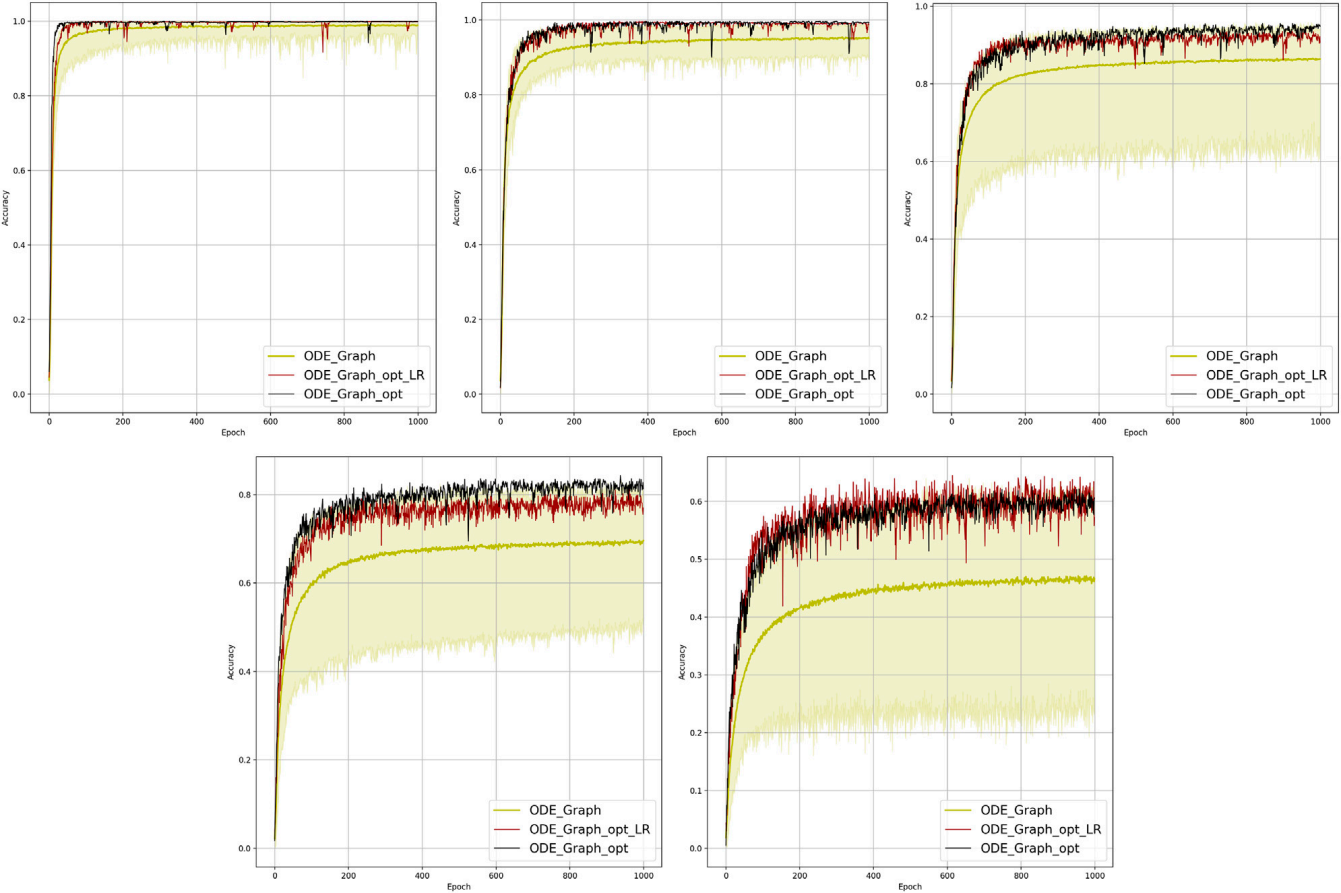
Comparison of the placement learning with the Graph ODE model for different observabilities. Sub-figures correspond to **(left-to-right and top-to-bottom)** 70, 40, 20, 10, and 5% of observability.

Results of the OP experiments are shown in Figures 4–9 for the LR, FFNN, GCNN, AlexNet, ODE Lin, and ODE Graph, respectively, each showing in sub-figure performances under 100, 70, 40, 20, 10, and 5% of nodes. Each sub-figure in the set corresponding to the advanced (all but LR) methods shows a comparative performance of 1) multiple IO-P samples, 2) OP-LR configuration found with LR-based training only, and 3) OP configuration found with LR-based pre-training and follow-up training on the corresponding model’s data.

### 3.4 Description of Software and Hardware

All the experiments were implemented on Python using Pytorch (Paszke et al., 2019) on Google Colab with an Intel Xeon CPU @ 2.20GHz and a 12GB NVIDIA Tesla K80 GPU.

## 4 Discussion

### 4.1 Detection of Failure: Discussion of Results

We have tested the performance of all the models introduced so far on the problem of fault detection on the experimental setup described in Section 3.1. We also included in this comparative study experiments with two other models introduced below. Specifically, in Section 2.2.1, we have adapted two Neural ODE models, both of which have a broader applicability to the case study of the fault detection in the case of the static observations.

Results of the 100 experiments with different randomly initialized parameters for each model are shown in Figure 2. Bold lines show mean accuracy curves for each model. We observe that, in general, the Linear ODE model performs better than other models. Also, all models outperform linear regression in low-observability regimes. Finally, our proposed models outperform the AlexNet-based model, which was suggested for the problem in the study by (Li et al., 2019). We also observed that the performance of the models depends dramatically on where the measurement (PMU) devices are placed. This observation-motivated material of Section 2.3 discusses the NN approach to the optimal placement of PMUs.

We expect that the NN methods described above will allow generalization/scalability to larger grids. Our ongoing exploration (work in progress, not reported in this manuscript) suggests that the number of NN parameters (and possibly layers) should grow linearly or faster with the grid size in order to result in a learning of a satisfactory quality.

### 4.2 Dynamic State Estimation Which Extrapolates: Discussion of Results

We observe that under full observability, the models which are the most physics informed, for example, DIRODENN and especially HNN, perform better than physics-agnostic models, of which the only linear one (LR) is the worst in performance. Systematic decrease in observability, from almost complete to modest, does not affect the qualitative assessment much. We interpret this preliminary conclusion (preliminary in view of the disclaimer above) as the confirmation of the general expectation that adding information about the structure of the power systems and especially about its dynamics helps to extrapolate, that is, in our context, represents part of the system where no samples were observed. On the other hand, when the observability becomes poor, it seems that the models which are from the middle of the pack (in terms of their use of the power system physics), such as the PINN and the Graph-ODENN, which are aware of the rather rough physical structure of the power system (and not about the details) are the winners of the performance competition. This suggests that planting too much of physics into the dynamic state estimation algorithm in the regime of low observability may also lead to a diminishing return.

### 4.3 Optimal Placement of Phasor Measurement Units: Discussion of Results

The experiments suggest that 1) finding optimal placement improves performance of the fault detection dramatically, 2) optimal placement of the PMU is a combinatorial optimization problem (of exponential complexity in the network size), which can be resolved efficiently (and, obviously, heuristically, i.e., approximately) using modern ML optimization software, 3) softening input and pre-training (with a fast but not accurate LR method) are steps which are critical for making the optimal placement algorithms efficient.

## 5 Conclusion

In this manuscript we first designed a sequence of NNs locating a faulty line. Different NN solutions were compared to each other at different levels of observability. The results suggest that NNs based on linear ODEs outperform other models at all the observability levels. Second, we proposed a sequence of the (power system) physics-informed NNs which allow us to predict the post-fault state. The results show that embedding this extra physical modeling in the NN helps; however, one also needs to be careful as constraining the learning too much (with the physical model) may lead to a diminishing return. Third, we designed an algorithm to improve PMU placement for better learning. Our methodology here is heuristics finding a satisfactory (but potentially suboptimal) solution.

We conclude by providing an answer to the question posed in the title. In this manuscript, we rely on synthetic experiments because mathematical derivations of the reduced models, for example, those represented by a NN, are not feasible at this stage. Our main point (which, one may say, is the main contribution of the manuscript) is not in providing solid guidance on which NN to use in each situation. Instead, we suggest (and show it on examples) that a researcher facing this challenge should be ready to test a number of different solutions. This custom search for the best (or simply good enough) NN solution depends on the following: 1) how much data/measurements are available? 2) How much explanation in terms of meaningful power system terms would we like to get? 3) How much extrapolation (to regimes unseen or purely represented in samples) is expected? We also suggest that as the problem becomes more challenging (fewer data and more explanations and extrapolations), we ought to rely more on embedding power system physics into the NN to succeed.

## Data Availability

The datasets presented in this study can be found in online repositories. The names of the repository/repositories and accession number(s) can be found below: https://github.com/AfoninAndrey/NNs-for-power-systems/tree/main/Datasets.

## Footnotes

1 Notice, in passing, that the two settings are most relevant to the post-fault control decisions made by the system operator, which are, however, not discussed in the manuscript directly.

2 Clarification on the terminology: we will use the terms “identification” and “localization” of the faulty line interchangeably for the task of discovering the faulty line within a power grid consisting of many lines. We do not focus in this manuscript on locating the position along the given line where the fault has occurred.

3 The model is universal in the sense that it stays the same regardless of the location where (c) has occurred. This setting is of a special relevance to the situations where PMU placement is relatively sparse and the faults considered are not severe.

4 In the following, we will combine *ϕ* and *ψ* in one set of parameters, where some of the parameters may be physical, that is, interpretable, and some, normally represented by an NN, can be physics blind.

5 In the DIRODENN scheme described below the *g* = 0 version of Eq. 16, ignoring power line resistance, was actually implemented.

6 The authors are grateful to Wenting Li for providing temporal, PST generated data.

7 All static models but AlexNet are tested in the regime of limited observability. This is because AlexNet is fine-tuned to the fixed size of the input, $x\left(0\right)\in R^{2\times 68}$, and adapting it to partial observability would require reconstructing the entire architecture of the NN. Moreover, AlexNet was not competitive in the case of the full observability.

8 In the cases of path-HNN_*ϕ*_ and path-DIRODENN_*ϕ*_, and according to the discussions above, some of the parameters will be physical and some physics-ignorant. Optimization in the respective version of Eq. 12 is over all the parameters.
